# Integrating Genetic and Gene Co-expression Analysis Identifies Gene Networks Involved in Alcohol and Stress Responses

**DOI:** 10.3389/fnmol.2018.00102

**Published:** 2018-04-05

**Authors:** Jie Luo, Pei Xu, Peijian Cao, Hongjian Wan, Xiaonan Lv, Shengchun Xu, Gangjun Wang, Melloni N. Cook, Byron C. Jones, Lu Lu, Xusheng Wang

**Affiliations:** ^1^Central Laboratory of Zhejiang Academy of Agricultural Sciences, Zhejiang Academy of Agricultural Sciences Hangzhou, China; ^2^Institute of Digital Agriculture, Zhejiang Academy of Agricultural Sciences Hangzhou, China; ^3^Institute of Vegetables, Zhejiang Academy of Agricultural Sciences Hangzhou, China; ^4^State Key Laboratory Breeding Base for Sustainable Control of Plant Pest and Disease, Zhejiang Academy of Agricultural Sciences Hangzhou, China; ^5^China Tobacco Gene Research Center, Zhengzhou Tobacco Research Institute of CNTC Zhengzhou, China; ^6^Department of Genetics, Genomics, and Informatics, University of Tennessee Health Science Center Memphis, TN, United States; ^7^Department of Psychology, University of Memphis Memphis, TN, United States; ^8^Department of Neurology, Affiliated Hospital of Nantong University Nantong, China; ^9^St. Jude Proteomics Facility, St. Jude Children’s Research Hospital Memphis, TN, United States

**Keywords:** alcohol, stress, network analysis, transcriptome, BXD mice strains, causality analysis

## Abstract

Although the link between stress and alcohol is well recognized, the underlying mechanisms of how they interplay at the molecular level remain unclear. The purpose of this study is to identify molecular networks underlying the effects of alcohol and stress responses, as well as their interaction on anxiety behaviors in the hippocampus of mice using a systems genetics approach. Here, we applied a gene co-expression network approach to transcriptomes of 41 BXD mouse strains under four conditions: stress, alcohol, stress-induced alcohol and control. The co-expression analysis identified 14 modules and characterized four expression patterns across the four conditions. The four expression patterns include up-regulation in no restraint stress and given an ethanol injection (NOE) but restoration in restraint stress followed by an ethanol injection (RSE; pattern 1), down-regulation in NOE but rescue in RSE (pattern 2), up-regulation in both restraint stress followed by a saline injection (RSS) and NOE, and further amplification in RSE (pattern 3), and up-regulation in RSS but reduction in both NOE and RSE (pattern 4). We further identified four functional subnetworks by superimposing protein-protein interactions (PPIs) to the 14 co-expression modules, including γ-aminobutyric acid receptor (GABA) signaling, glutamate signaling, neuropeptide signaling, cAMP-dependent signaling. We further performed module specificity analysis to identify modules that are specific to stress, alcohol, or stress-induced alcohol responses. Finally, we conducted causality analysis to link genetic variation to these identified modules, and anxiety behaviors after stress and alcohol treatments. This study underscores the importance of integrative analysis and offers new insights into the molecular networks underlying stress and alcohol responses.

## Introduction

Stress is a physical, mental or emotional response to events that disrupt normal homeostasis (Lazarus et al., 1952; Selye, 1998). Stress is one of the contributing causes to many mental disorders, such as depression and anxiety. A major component of the stress response system is activation of the hypothalamic–pituitary–adrenocortical (HPA) axis (Smith and Vale, 2006). HPA axis dysregulation is postulated to significantly influence motivation for alcohol behaviors, including alcohol exposure and withdrawal (Koob and Le Moal, 2001); however, the underlying molecular mechanisms of the interplay between stress and alcohol remain unclear.

Over the past decade, a number of genetic loci involved in either stress or alcohol, or combined responses have been uncovered by genetic linkage and association studies (Gelernter et al., 2014) and genome sequencing analyses (Clark et al., 2017). For example, genetic linkage using quantitative trait locus (QTL) mapping identified a tyrosine phosphatase (*Ptp4a1*) and a transcription factor (*Phf3*) as candidate genes for stress-alcohol interactions (Cook et al., 2015). These same genes have also been nominated as candidate genes for alcohol dependence in a human genetic association study (Zuo et al., 2011). We further confirmed that *Ptp4a1* is a candidate gene in stress and alcohol responses (Baker et al., 2017) by using differentially expressed (DE) genes and QTL analysis in BXD recombinant inbred (RI) strains. In addition, we identified a sequence variant in a circadian rhythm gene, *Per3*, and associated it with stress and alcohol responses by integrating expression QTL (eQTL) and phenotype QTL analyses (Wang et al., 2012). Although many individual genes have been identified by various genetic studies, the interaction of these genes at the molecular network level has not been extensively explored.

Weighted Gene Co-Expression Network Analysis (WGCNA) for gene expression has been successfully applied to identify functional modules implicated in diseases (Voineagu et al., 2011). In recent years, several studies have applied the WGCNA approach to successfully identify transcriptional networks associated with alcohol-related studies, including alcohol consumption in liver of the HXB/BXH RI strains (Saba et al., 2015; Hoffman et al., 2018) and in different brain regions of the BXD RI strains (Vanderlinden et al., 2013), consumption and withdrawal in striatum of the BXD F2 strains (Metten et al., 2014) and of the heterogeneous stock mice (Iancu et al., 2013), alcohol dependence in human brain (Ponomarev et al., 2012; Farris et al., 2015). The approach has extended to identify mRNA and miRNA co-expression modules in a matched human alcohol dependence case-control postmortem samples (Mamdani et al., 2015). Compared to differential expression analysis, the co-expression network approach can avoid multiple testing problems (Langfelder and Horvath, 2008). Co-expression modules between different genetic or environmental conditions can be compared to examine module preservation and specificity (Miller et al., 2010). Combining co-expression gene modules with protein-protein interaction (PPI) could further identify functional sub-networks that are potential involved in a disease (Oldham et al., 2008). Recently, structural equation model (SEM) is often used (Vermunt and Magidson, 2014) to identify causal relations from genetic variation to co-expression modules, and anxiety phenotypes.

In this study, we apply co-expression network analysis to transcriptomes of the BXD RI mice under stress and alcohol responses. The co-expression analysis identifies 14 modules for combined four conditions and characterizes four expression patterns. PPIs are mapped to co-expression modules to define four functional subnetworks. We further perform modular specificity analysis between stress, alcohol and stress-induced alcohol. Finally, we performed SEM-based causative analysis to link genetic variation to expression modules and anxiety phenotypes.

## Materials and Methods

### BXD RI Mice and Treatments

The BXD strains were raised at the University of Tennessee Health Science Center and were between 60 and 95 days of age. A total of 41 BXD mouse strains and parental strains of adult male and female mice (Supplementary Table S1; an average of two mice per strain per group; *n* = 211) were used for phenotyping and expression profiling following exposure to stress, treatment with ethanol, or their combination. Within each strain, animals were separated into four conditions:

- Group 1, acute stress (RSS: Restraint stress followed by a saline injection): These animals were subjected to acute restraint in a ventilated 50 ml centrifuge tube for 15 min.
- Group 2: acute ethanol (NOE: No restraint stress and given an ethanol injection): These animals received a 1.8 g/kg i.p. injection of ethanol (12.5% v/v).
- Group 3: stress plus ethanol treatment (RSE: Restraint stress followed by an ethanol injection): For these animals, ethanol injections were administered immediately after the restraint exposure received saline injections at the same volume in the ethanol group.
- Group 4: Control (NOS: No restraint stress): These animals received saline injections (isovolumetric to the ethanol dose), but were not exposed to stress or ethanol injections.

All procedures involving animals were approved by the Animal Care and Use review boards of The University of Tennessee Health Science Center and The University of Memphis. More detailed information about BXD strains for each group is available at GeneNetwork:

- NOS: http://genenetwork.org/webqtl/main.py?FormID=shar inginfo&GN_AccessionId=814
- RSS: http://genenetwork.org/webqtl/main.py?FormID=shar inginfo&GN_AccessionId=815
- NOE: http://genenetwork.org/webqtl/main.py?FormID=shar inginfo&GN_AccessionId=816
- RSE: http://genenetwork.org/webqtl/main.py?FormID=shar inginfo&GN_AccessionId=817

### Behavioral Phenotypes

The phenotypes were measured for four treatment groups: NOS, RSS, NOE and RSE. Five minutes after injection with saline or ethanol, the mice were placed in an elevated zero-maze, which is commonly used to measure the anxiety level and activity of mice, for 10 min and their behavior was monitored. The behavior of the mice was measured using two phenotypes over three periods of time. The first phenotype was activity count (ACTCNT) calculated as the average number of beam breaks of infrared sensors in closed quadrants per second over 0–5 min (ACTCNT0–5), 5–10 min (ACTCNT5–10), and 0–10 min (TOTAL ACTCNT) of the time spent in the maze. The second phenotype was the percentage of time spent in open quadrants (OPEN) over 0–5 min (OPEN0–5), 5–10 min (OPEN5–10), and 0–10 min (TOTAL OPEN). A summary of behavioral phenotypes is provided in Supplementary Table S2. Behavioral testing was carried out at the University of Memphis.

### RNA Isolation and Microarray Experiments

Four hours after the initial injection, mice were sacrificed by cervical dislocation and brains were removed (Wang et al., 2012). Hippocampal dissection was conducted and hippocampal RNA was isolated according to manufacturer’s protocol using RNA STAT-60. Gene expression for all groups was examined by microarray analysis as previously described (Wang et al., 2012; Baker et al., 2017). Hippocampal gene expression was analyzed using Illumina v6.1 microarrays, according to the manufacturer’s protocol^1^. All data were normalized using the rank invariant method and background subtraction protocols outlined by Illumina in the BeadStation software. All four datasets were normalized by Illumina Rank Invariant method and then performed Log2 Transformed and Z-score. Batch effects were removed using analysis of variance (ANOVA). Strain and sex assignments were verified and corrected.

### Genotype and Microarray Annotation

Genotypes and phenotypes were downloaded from GeneNetwork^2^. The genotype data include 3811 markers. The detailed information about genotypes and phenotypes can be found in our previous study (Wang et al., 2016). An annotation file available on the GeneNetwork Data Sharing Zone^3^ was used to determine the genes and genome locations associated with the 46,643 unique probe sequences in the Illumina Mouse 6.1 array.

### Analysis of Identified Variable Genes

A total of 16,578 probe sets on the Illumina v6.1 microarray were used to interrogate gene expression across all four conditions, each with on average 45 BXD strains. To detect variable expression genes for network analysis, we calculated the coefficient of variation (CV) for each gene across all conditions and BXD strains. The distributions of CV were fitted by two mixed normal distributions using an expectation–maximization (EM) algorithm to define variable genes. The left side of the normal distribution was defined as representing invariable genes, whereas the right side of the distribution was considered to represent variable genes.

### Gene Co-expression Analysis

We constructed weighted gene co-expression networks based on variable genes using the WGCNA package in R (Langfelder and Horvath, 2008). The product was a weighted adjacency matrix that provided continuous connection strength ([0, 1]) based on the β parameter for each condition to meet the scale-free topology criterion. Subsequently, the co-expression matrix and the topological overlap matrix (TOM) were constructed. For TOM, we assessed the interconnectedness of two genes by the degree of their shared neighbors across the global network. We detected the gene modules by average linkage hierarchical clustering for each group. The intra-modular connectivity of each gene was also computed using the intra-modular connectivity function in R. The module eigengene (ME) is the first principal component of a given module, and it was used to evaluate the module membership, which assesses the importance of genes in the network.

### Module Preservation Statistics

To assess modular preservation and specificity of any two modules from two different conditions, we computed the number of shared genes between the two modules and then used Fisher’s Exact Test to calculate the significance. *p* < 0.01 was used as a threshold.

### Protein-Protein Interaction Network and Subnetwork Construction

PPI networks provide valuable information toward understanding cellular functions and biological processes. In this study, PPI networks were constructed based on the protein interaction information retrieved from STRING (version 10; Search Tool for the Retrieval of Interacting Genes/Proteins). To define subnetworks, we first only selected those interactions having an interaction score greater than 700 (high-confidence). These highly confident interaction in PPI network were then mapped to the co-expression modules to examine whether any common interactions were found in both networks. Those common interactions were defined as subnetworks in this study. The subnetworks and the linker genes were visualized using Cytoscape software (Kohl et al., 2011).

### Causative Analysis to Link Genotype to Phenotype

Structural equation modeling (SEM) was used to infer causality among genetic variants (SNP markers), co-expression modules and phenotypes. The Network Edge Orienting (NEO) method uses SEM to estimate the probabilities for each of the three relationships. The NEO was used to evaluate causality as previously described (Aten et al., 2008). Briefly, the NEO uses phenotype, transcriptome and genotype information to infer a causal link between genotype, module and phenotype. We applied this analysis to a phenotype, genotypes that had the phenotypic QTL with the highest likelihood score, genes in each module associated with the phenotype. Default parameters for NEO were used as originally described (Aten et al., 2008) in which NEO estimates the likelihoods of all local SEMs and returns a Local Edge Orientation (LEO) likelihood score between genotypes, genes and phenotypes. To make a determination of edge orientation, we only examined “forward” (genotype affects gene, and then phenotype). The LEO score is defined by log10 of dividing the model *p*-value for genotype→gene→phenotype (Model 1) by the *p*-value of the best fitting alternative models (Model 2: genotype→phenotype→gene; Model 3: gene← genotype→phenotype; Model 4: genotype→gene←phenotype; Model 5: genotype→phenotype←gene). For example, an LEO score of 1 indicates that genotype →gene→phenotype fits the data ten times better than any competing model when the fit is measured using the *p*-value. An LEO score of 0.5 was used as a threshold. The more positive the score, the stronger the evidence.

### Over-Representation Analysis

We used Fisher’s Exact Test to measure the statistical over-representation of cell type *i* in co-expression module *j*. *p-values* are computed from a 2 × 2 contingency table comprised of: (1) the number of genes in both module *i* and cell type *j*; (2) the number of genes in module *i* but not in cell type *j*; (3) the number of genes in cell type *j* but not in module *i*; and (4) the number of genes in neither module *i* nor cell type *j*. The test statistic is implemented in the R software package. *p* value < 0.05 was considered as statistical significance.

The over-representation of gene ontology (GO) functional terms for each module was conducted by the “GOenrichmentAnalysis” function in the WGCNA package (Langfelder and Horvath, 2008). This function uses the Fisher’s Exact Test on overlaps of GO terms, including their GO offspring, and modules. The top enriched GO term was selected as representation for each module. The mouse was selected as the organism in the function.

### Statistical Analysis for Expression Pattern

A standard linear one-way ANOVA was performed each expression pattern. For each expression pattern, an average of the expression of all genes in each module (*E*_ij_), which is similar to module eigenvalue, was used as the dependent variable. The effect examined for each expression pattern was treatment/condition with four levels (NOS, RSS, NOE and RSE). The following linear model was used:

$$E_{\text{ij}}=\mu +T_{\text{i}}+\varepsilon_{\text{ij}}$$

where *u* is the grand mean of the observations; *T*_i_ is the effect of the *i*th treatment; *ɛ*_ij_ is random error which is distributed normally with mean zero, *ɛ*_ij_ ~ *N*(0,*σ*^2^). *p*-value was generated for each expression pattern using a standard F-test.

## Results

**Figure 1:**
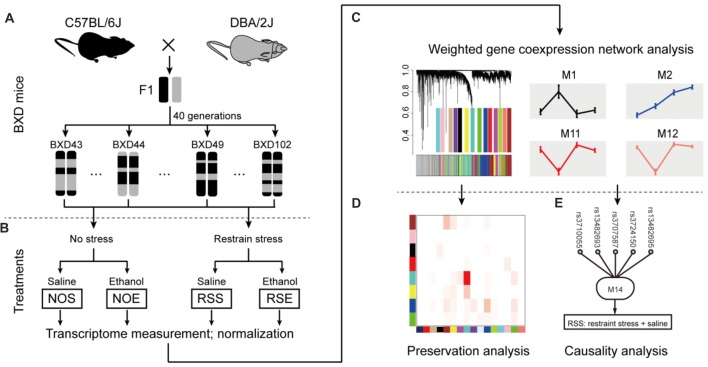
Diagram showing network-based data analysis. **(A)** The expression data were generated from the BXD recombinant inbred (RI) mice. **(B)** The transcripts of these four conditions were compared and analyzed. **(C)** Weighted gene co-expression network analysis by WGCNA. **(D)** Conservative analysis of gene modules under experimental conditions. **(E)** Causal analysis.

**Figure 2:**
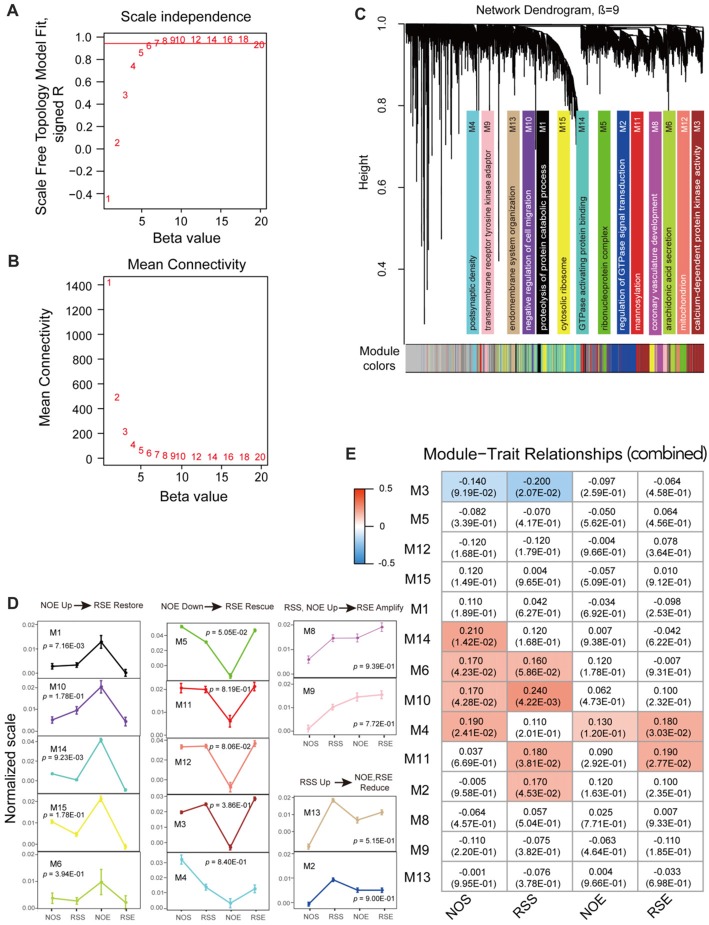
Co-expression module analyses. **(A)** The soft thresholding index *R*^2^ (y-axis) as a function of different powers β (x-axis). **(B)** The mean connectivity (y-axis) is a strictly decreasing function of the power β (x-axis). **(C)** Fourteen co-expression modules identified from the combined dataset. WGCNA cluster dendrogram groups genes (*n* = 6413) measured across BXD Hippocampus into distinct gene modules (M1–14) defined by dendrogram branch cutting. These modules were significantly enriched for gene ontologies linked to discrete cellular functions and/or organelles in the brain. Genes that did not belong to any modules were housed in the gray modules. The gray gene modules were ignored in this study. **(D)** Four expression patterns. Four expression patterns were found: up-regulation in No restraint stress and given an ethanol injection (NOE) but restoration in Restraint stress followed by an ethanol injection (RSE) (NOE Up→RSE Restore); down-regulation in NOE but rescue in Restraint stress followed by an ethanol injection (NOE Down→RSE Rescue); up-regulation in both Restraint stress followed by a saline injection (RSS) and NOE, and further amplification in RSE (RSS, NOE Up→RSE Amplify); up-regulation in RSS but reduction in NOE and RSE (RSS Up→NOE, RSE Reduce). One-way analysis of variance (ANOVA) was used to determine the conditions which are significantly different from each other for each expression pattern. ANOVA *p*-values are indicated in each pattern. The error bar represents standard error of the mean (SEM). **(E)** Heat maps of Pearson correlation and *p*-value between modules and traits. Each cell represents the correlation coefficient (and *p*-value) computing from correlating module eigengenes (MEs) (rows) to traits (columns). Only those correlations with |*p*| < 0.1 are shown.

To elucidate molecular networks underlying stress, alcohol and stress-induced alcohol relationships, we used an integrative approach that combines gene co-expression, PPI networks, and causative analysis to identify genes associated with alcohol and stress responses. Gene expression in the hippocampus of 41 BXD RI mice was measured by microarray for four conditions, including exposure to ethanol (NOE), stress (RSS), the combination of both (RSE), and control without any treatment (NOS; total sample number *n* = 211; an average of 2 mice per strain per group; Supplementary Table S1; Figures 1A,B). The integrative approach involved three steps: (1) constructing weighted gene co-expression networks of the stress and alcohol response conditions (Figure 1C); (2) analyzing the specificity of each network between four conditions (Figure 1D); and (3) identifying causal genes underlying each network that regulates expression of genes and modulates stress and alcohol responses (Figure 1E).

### Weighted Gene Co-expression Networks of Stress and Alcohol Responses

To gain insight into the functional organization of the transcriptomes from four conditions (NOS, RSS, NOE and RSE), we constructed gene co-expression networks using highly variable genes (see “Materials and Methods” section). Four-stepwise criteria were used to produce a list of highly variable genes across the BXD RI strains and four conditions. First, we measured the reproducibility between replicates for four conditions. Expression of each condition showed a high reproducibility, with an average coefficient of determination (*R*^2^) of 0.9993 (Supplementary Figure S1). Second, the variability of the expression level of each gene across BXD strains was determined by calculating the CV. Third, variable genes were decomposed by using the EM algorithm. Finally, genes with average expression value less than 8 were removed. With these four steps, we detected an average of 6569 highly variable genes for each condition (Supplementary Figure S2). The variable genes across all four conditions were merged into a combined dataset (*n* = 6413) for subsequent analyses (Supplementary Table S3).

We used WGCNA program to construct gene co-expression networks for a combined data set that is generated by combining expression data of four conditions. We first determined the soft-thresholding power (β = 9) in accordance with the scale-free topology criterion (Figures 2A,B). By using this soft thresholding power, we identified a total of 14 modules (Supplementary Table S4; Figure 2C). The module size (i.e., the total number of genes in a module) varies significantly, ranging from 104 genes in module M4 to 1047 genes in module M14 (Supplementary Tables S5, S6). Similarly, we performed co-expression network analysis for each condition (Supplementary Figures S3, S4). We detected a total of 72 modules, ranging from eight modules in RSS to 21 in RSE (Supplementary Table S4). The modules for each condition also vary in size. For example, module M1 in NOE condition contains as many as 1405 genes, whereas module M13 only contains 53 genes.

To investigate pattern changes in expression among conditions, we summarized the 14 detected modules into four patterns with respective to up- or down-regulation in alcohol or stress response for the combined dataset. The four patterns include up-regulation in NOE but restoration in RSE (pattern 1: NOE Up→RSE Restore); down-regulation in NOE but rescue in RSE (pattern 2: NOE Down→RSE Rescue); up-regulated in both RSS and NOE, and amplified in RSE (pattern 3: RSS, NOE Up→RSE Amplify); up-regulated in RSS, reduced in NOE and RSE (pattern 4: RSS Up→NOE, RSE Reduce). One-way ANOVA and *post hoc* Tukey’s test were utilized to determine the conditions which are significantly different from each other for each expression pattern (Supplementary Table S7). The first expression pattern includes five modules, including modules M1 (*p-value* < 0.01), M6 (*p-*value < 0.40), M10 (*p-*value < 0.18), M14 (*p-*value < 0.01), and M15 (*p-*value < 0.18; Figure 2D; left panel). Genes in this pattern exhibit strong up-regulation in NOE but restoration in RSE, suggesting that these genes are activated by alcohol but counterbalanced by the interaction of alcohol and stress. The M1 module includes a cluster of γ-aminobutyric acid receptor subtype A (GABA_A_) genes (*Gabra2*, *Gabra1* and *Gabrb1*). A neurotropic factor, *Creb3*, encoding cAMP response element-binding protein, was also found in the module M1.

The second pattern includes five modules, including M3 (*p*-value < 0.39), M4 (*p*-value < 0.84), M5 (*p*-value < 0.06), M11 (*p*-value < 0.82) and M12 (*p*-value < 0.09; Figure 2D; middle panel). Genes in this pattern exhibit down-regulation in NOE but rescue in RSE. For example, the module M11 contains the *Aldh2* gene which is associated with alcohol metabolism. Additional genes, *Dnm1* (dynamin-1) and *Comt (*Catechol-o-methyltransferase), in module M3 are related to neurotransmission. *Dnm1* is involved in endocytic- and energy-related pathways in alcohol treatment, while *Comt* is important in the metabolism of catecholamines (including dopamine, epinephrine and norepinephrine). The third pattern includes two modules, M8 (*p-*value < 0.94) and M9 (*p-*value < 0.78; Figure 2D; upper-right panel). Genes in this pattern exhibit up-regulation in both RSS and NOE, and further amplification in RSE. The fourth pattern includes 2 modules, M2 (*p-*value < 0.90) and M13 (*p-*value < 0.52; Figure 2D; lower-right panel). Many genes in these modules are associated with intracellular signaling cascades. For example, module M2 contains the *Grin2* gene, which is related to glutamate receptor signaling.

We then summarized the expression levels of each module by the first principal component (ME), and assessed the extent to which the modules were related to anxiety phenotypes associated with the four conditions (Supplementary Table S2). For the 14 modules produced by the combined dataset, the module M2 shows significant correlation with the RSS phenotype (*p* = 0.05), and the module M11 shows significant correlation with the RSS phenotype (*p* = 0.04) and the RSE phenotype (*p* = 0.03; Figure 2E). Consistent with expression patterns as shown above, the module M2 is one that is up-regulated in the RSS condition, and the module M11 is up-regulated in both RSS and RSE conditions compared to NOE (Figure 2D). We also examined the relationship between modules from individual expression datasets and the four phenotypes. For both the NOS and RSS modules, none of the modules were significantly correlated with their respective phenotypes (Supplementary Figures S5A,B). Interestingly, module M11 in NOS shows a significant correlation with RSE phenotype (*p* = 3.6 × 10^−3^). For the 13 NOE modules, the module M1 shows the most significant correlation with its phenotype (*p* = 5.6 × 10^−3^; Supplementary Figure S5C). The expression pattern of the module M1 is also up-regulated in NOE compared to other conditions (Figure 2D). Four of the RSE modules (M2, M5, M13 and M20) were highly correlated with its phenotype (*p* < 5 × 10^−2^; Supplementary Figure S5D).

Because the hippocampus is a heterogeneous structure, we also determine modules enriched in genes expressed by different cell types. We evaluated the enrichment of each module with nine hippocampus cell types, which were previously generated from mice hippocampus using single cell RNA sequencing, including ependymal, oligodendrocyte, microglia, CA1 pyramidal, interneuron, endothelial, S1 pyramidal, astrocyte and mural (Zeisel et al., 2015). We observed four modules (i.e., M5, M6, M14 and M15) are statistically significantly enriched for microglia, ependymal, astrocyte, and endothelial, respectively (*p* < 0.05; Fisher Exact test; Supplementary Table S8). The module, M5, was enriched with microglia-specific transcripts (*p* < 4.9 × 10^−2^), including *Mbnl1*, *Fau*, *C1qa*, *Ctsz* and *0610031J06Rik*. The module, M6, was also enriched for ependymal markers (*p* < 4.5 × 10^−2^), including *Ascc1*, *Smad5* and *1700029J07Rik*. Twenty-one astrocyte genes were predominantly enriched in M14 (e.g., *Adk*, *Ckb* and *Akt2*; *p* < 3.9 × 10^−2^). Finally, 14 endothelial markers were over-represented in the M15 module (e.g., ribosomal genes *Rps6*, *Rps18*, *Rpl9* and *Rpl23*).

### Functional Subnetworks Identified by Relating Protein Interaction Networks and Co-expression Modules

**Figure 3:**
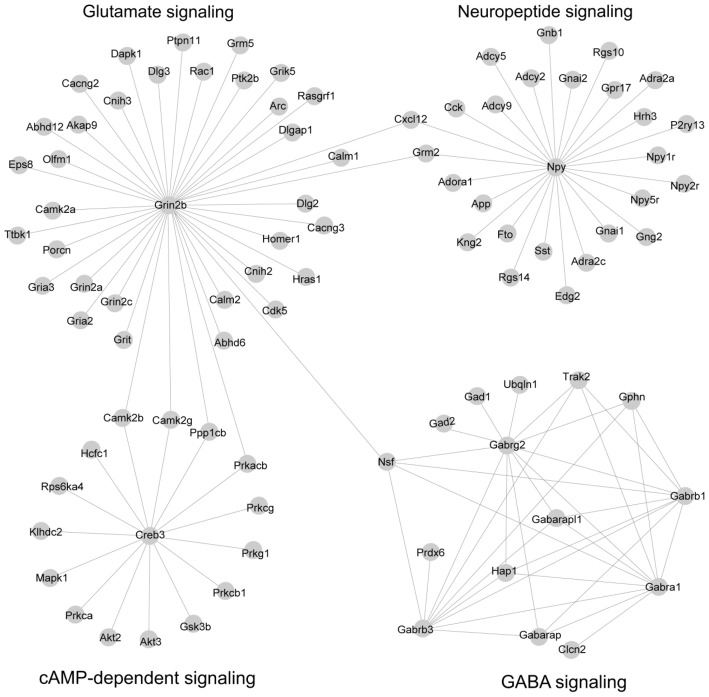
Subnetworks were constructed by combining co-expression network and protein-protein interaction (PPI) network. A total of 14 co-expression modules identified by co-expression analysis were superposed with PPI networks from the STRING database, leading to four subnetworks. The network was visualized by Cytoscape software.

We reasoned that genes in both PPI networks and co-expressed networks are more likely to share common molecular functions (Brown and Botstein, 1999; Zhang and Horvath, 2005). By superimposing PPI networks from the STRING database to 14 co-expression modules, we identified four subnetworks in which the genes are highly inter-connected at both the transcription level (co-expression network) and the protein level (PPI interaction network; Figure 3; Supplementary Table S9).

Subnetwork 1 is mainly involved in γ-aminobutyric acid (GABA) signaling. The subnetwork includes six GABA receptors, *Gabra1*, *Gabrb1*, *Gabrb3*, *Gabrg2*, *Gabarap* and *Gabarapl1*; GABA receptors were first identified as a target of alcohol (Martz et al., 1983). It also includes two GABA decarboxylase enzymes (*Gad1* and *Gad2*) which catalyze the synthesis of GABA. These two enzymes, *Gad1* and *Gad2*, have been implicated in acute alcohol withdrawal severity (Buck et al., 1997), alcohol preference (Phillips et al., 1998) and alcohol-induced locomotion (Demarest et al., 1999) in the BXD RI strains. Finally, this subnetwork includes three genes that are highly related to GABA synapses (*Nsf*, *Gphn* and *Trak2*); GABA synapses contribute to many of alcohol behaviors, including dependence and withdrawal (Ariwodola and Weiner, 2004). This subnetwork, presumably, is a subnetwork implicated in alcohol responses.

Subnetwork 2 is largely associated with glutamate signaling, with a hub gene of *Grin2b*, an N-methyl-D-aspartate receptor (NMDA) receptor. Glutamate signaling is mediated through the activation of two families of transmembrane receptors: G-protein coupled receptors (mGluRs) and ligand-gated ion channels including NMDA, AMPA and kainate receptors (Traynelis et al., 2010). This subnetwork includes two additional NMDA receptors (*Grin2a* and *Grin2c*), three AMPA receptors (*Gria2*, *Gria3* and *Grik5*), and metabotropic glutamate receptor (*Grm5*). The NMDA and AMPA receptors are well-known for their involvement in alcohol’s effects (Ron and Wang, 2009; Salling et al., 2016). The *Grm5* gene has been linked to alcohol dependence in both humans and mice (Ceccarini et al., 2017). In addition, epidermal growth factor receptor kinase substrate 8, *Eps8*, is localized part of the NMDA receptor complex, is a regulator of actin dynamics and has been shown to increase ethanol consumption in mice (Offenhäuser et al., 2006). Overall, this module is highly related to alcohol response.

Subnetwork 3 is a neuropeptide network. Neuropeptides act as neuromodulators in the brain and in the autonomic nervous system. This subnetwork includes neuropeptide Y (*Npy*) and three *Npy* receptor genes (*Npy1r*, *Npy2r* and *Npy5r*). Several lines of evidence in both human and animal studies suggest that variations in these four genes are associated with alcohol dependence as well as alcohol withdrawal symptoms (Ehlers et al., 1998; Wetherill et al., 2008; Kokare et al., 2017). In addition, G-protein alpha (*Gna1*, *Gnal2*, *Gnb1* and *Gng2*) and its regulators (*Rgs10* and *Rgs14*) are included in this subnetwork. The involvement of these genes in both alcohol and stress responses is well known (Baker et al., 2017).

Subnetwork 4 is associated with cAMP-dependent signaling. The cAMP signaling pathway has been strongly implicated in both anxiety-like and alcohol-drinking behaviors. This pathway is activated by the protein kinase C (PKC) complex, Protein kinase B (AKT), and Ca^2+^/calmodulin-dependent protein kinase II (CAMK2). In this subnetwork, we found four PKC genes (*Prkca*, *Prkcb1*, *Prkcg* and *Prkg1*) and two protein kinase B genes (*Akt2* and *Akt3*), and two CAMKII genes (*Camk2b* and *Camb2g*). We also identified glycogen synthase kinase 3 (*Gsk3*), which inhibits the cAMP pathway in this subnetwork and has been linked to mechanisms associated with stress, mood regulation and the effects of antidepressants (Pavlov et al., 2017). Both kinases, *Akt* and *Gsk3*, play critical roles in ethanol-induced cognitive impairment (Wang et al., 2016). This subnetwork is believed to be linked to the interaction of alcohol and stress responses.

### Module Specificity Between Networks of the Stress and Alcohol Responses

**Figure 4:**
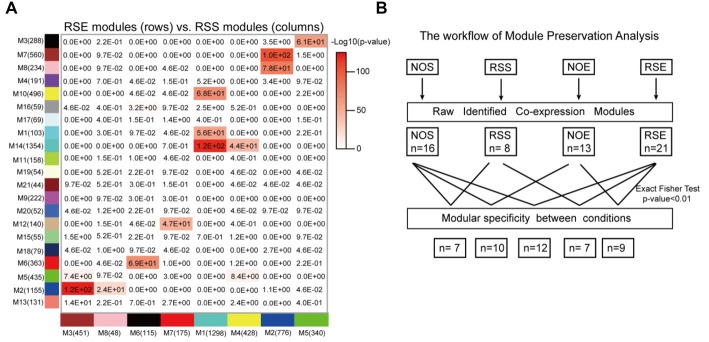
Module specificity and preservation. **(A)** Perseveration of co-expression networks between RSE and RSS. Each square in the graph represents the degree of overlap between two modules. The number in the cell represents the probability of module preservation between two conditions. A hypergeometric two-tailed Fisher’s exact test was used to determine the probability. **(B)** Summary of module preservations for all four conditions.

Differences among network organization could provide a basis for better understanding the molecular processes underlying alcohol and stress responses. To assess the modular differences between four conditions on a module-by-module basis, we first calculated the modular preservation, which is defined as whether genes in a module in one condition are enriched in any modules in the other conditions (see “Materials and Methods” section). The preservation was computed by Fisher’s exact test and a *p*-value of 0.01 was applied. We observed that 12 modules (Figures 4A,B) are significantly preserved between RSE and RSS. We identified a module M21, containing 44 RSE-specific genes (Supplementary Table S10), of which nine genes (*Gabra3*, *Slc6a11*, *Slc17a6*, *Fgf12*, *Cacna1h*, *Prkcd*, *Diras1*, *Adcy8* and *Vangl1*) were found to be associated with either alcohol, or stress responses, or both. *Gabra3* and *Slc6a11*, are GABA receptors and transporters respectively, which are known to be involved in alcohol responses. For example, expression levels of vesicular glutamate transporter genes including *Slc17a6* can be reduced by alcohol exposure (Flatscher-Bader et al., 2008). This result suggests a possible molecular link between Module M21 and the interaction between alcohol and stress responses.

Similarly, we examined module specificity between NOS and NOE, between NOS and RSS, and between NOE and RSE (Supplementary Figure S6). We observed two NOS-specific modules: M6 and M14, when comparing NOS to the other three conditions (i.e., RSS, NOE and RSE), suggesting that genes in these two modules were dysregulated either in alcohol or stress response. M6 contains 68 genes, including *Crhbp*, *Dpysl3*, *Mekk3* and *Msk1*. *Crhbp* and *Dpysl3* are known to modulate stress responses in genetic mouse models (Ketchesin et al., 2017). In addition, this module also contains eight histone cluster 1 and cluster 2 genes, including *Hist1h2af*, *Hist1h2ag*, *Hist1h2ah*, *Hist1h2ai*, *Hist1h2ak*, *Hist1h2an*, *Hist1h2ao* and *Hist2h2ac*, suggesting this module may be involved in epigenetic regulation. M14 contains 76 genes, including *Slc12a2* and two insulin-like growth factor binding proteins (IGFBP2 and IGFBP7). Upregulation of *Slc12a2* induces loss of GABAergic inhibition of stress-induced corticotrophin-releasing hormone levels (Gao et al., 2017). IGFBP7 treatment was also associated with strong activation of the stress associated p38 MAPK pathway (Benatar et al., 2012).

### Causative Analysis for Co-expression Modules

**Figure 5:**
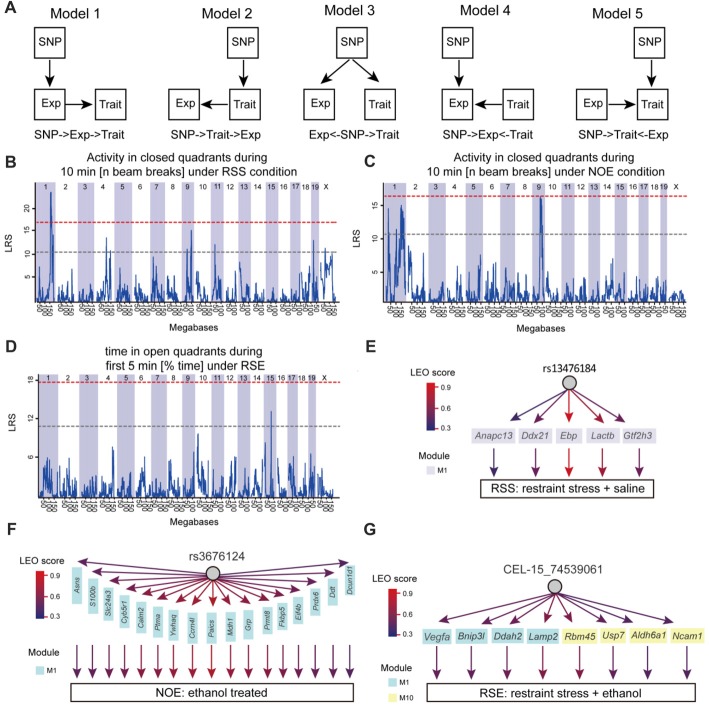
Causative analysis. **(A)** Schematic diagram showing SNP→Module→Phenotype causality analysis. Five possible Single Anchor Models were shown. **(B–D)** Quantitative trait locus (QTL) mapping for three conditions, including No restraint stress (NOS), RSS and RSE. **(E–G)** The causal network was constructed by SEM methods for NOE, RSS and RSE.

Finally, to address whether the identified modules and genes within the module are likely to cause the phenotypic outcomes, the SEM method (Figure 5A) was used to infer causal relationships between genetic variation, gene expression, and 24 behavioral phenotypes generated for this study (Supplementary Table S11). Here, we used three SNP markers (*rs13476184*, *rs3676124* and *CEL-15_74539061*) that showed the highest Likelihood Ratio Statistics (LRS) for three behaviors of our studied conditions (RSS: Activity in closed quadrants during 10 min under RSS condition; NOE: Activity in closed quadrants during 10 min under NOE condition; and RSE: Time in open quadrants during first 5 min under RSE), respectively (Figures 5B–D). We performed causality tests for these three genetic markers, genes in all identified modules, and all behavioral phenotypes using the NEO algorithm. Genes causally affecting ≥1 phenotype were ranked by their maximum scores. A causality Local SEM-based Edge Orienting (LEO) score threshold of 0.5, equivalent to a 3.2-fold (10^0.5^) higher probability compared with any other model, was used. Thus, the NEO provides a way to prioritize genes that may cause the behavioral phenotypes.

Figure 5 shows the results of NEO analysis for the three genetic markers that are mapped to RSS, NOE and RSE. For all modules identified in the expression of RSS, we identified five genes within RSS module M1 that link the *rs13476184* marker and RSS behavioral phenotype. The five genes include *Anapc13*, *Ddx21, Ebp*, *Lactb* and *Gtf2h3* (Figure 5E), of which the *Ebp* gene shows the highest Local Edge Orienting (LEO) score. The *Ebp* gene encodes an integral membrane protein that functions as a key enzyme in restraint stress (Jangra et al., 2016). Similarly, we identified a link between the NOE genetic marker *rs3676124*, 17 genes in NOE module 1, and the NOE behavioral phenotype (Figure 5F). Among the 17 NOE module genes, seven (*Prdx6*, *Grp*, *Cyb5r1*, *Ddt*, *S100b*, *Calm2* and *Ptma*) show *cis*-regulation with a fixed cutoff of LRS > 15 (Likelihood ratio (LOD) > 3) in NOE eQTL analysis. Note that the fixed cutoff may not met genome-wide statistically significant level for all eQTLs. The *Prdx6* gene, which is a member of the peroxiredoxin family of antioxidant enzymes, had the highest LRS (23.4). Previous studies have shown that *Prdx6* plays a role in alcohol metabolism (Roede et al., 2009). For RSE, we identified four genes (*Vegfa*, *Bnip3l*, *Ddah2* and *Lamp2*) in module M1, and four genes (*Rbm45*, *Usp7*, *Aldh6a1*, *Ncam1*) in module M10 that connect the genetic marker *CEL-15_74539061* and the RSE behavioral phenotype (Figure 5G). *Aldh6a1* is a member of the aldehyde dehydrogenase family that is thought to play a major role in alcohol metabolism (Quertemont, 2004). For all three conditions, we observed that a genotype influences expression of multiple genes, indicating potential pleiotropic effects. In summary, we postulate that *Ebp, Prdx6, and Aldh6a1* are potential causal genes for RSS, NOE and RSE, respectively.

## Discussion

In this study, we identified molecular networks mediating alcohol and stress responses, as well as their interaction in the hippocampus of mice using a systems genetics approach that integrates co-expression network analysis, protein-protein network, and causality analysis. We identified 14 modules for the combined dataset. By combining co-expression and PPI networks, four functional subnetworks were found. We also observed two NOS-specific modules and one RSE-specific module. We investigated potential causal genes involved in alcohol and stress responses using the SEM algorithm. Overall, this comprehensive analysis allows for the identification of molecular networks and causal genes that likely mediate stress and alcohol responses.

Over the past decade, forward and reverse genetics approaches have been widely used in the study of complex traits, such as alcohol consumption, alcohol administration and stress response. Although these traditional genetics approaches identified many genes underlying complex traits, they provide little information on molecular networks linking genotypes to specific phenotypes. In contrast to traditional genetic studies, we first constructed molecular modules through co-expression analysis and identified potential causal genes within the modules. The advantage of this network-based approach is that it provides a clear picture of the connection between genotype and phenotype. The analysis brought us to the identification of candidate modules, subnetworks and causative genes.

In the previous study (Baker et al., 2017), they identified 15 differentially expressed genes (DEGs) after exposure to acute stress (RSS vs. NOS), 243 DEGs after exposure to ethanol (NOE vs. NOS), and 70 DEGs after exposure to stress and ethanol combination, respectively. They further found five RSS DEGs, 38 NOE DEGs and 27 RSE DEGs that highly correlate with phenotypes within each group. Among these, 16 DEGs were found in our detected modules, including 14 DEGs in six NOS modules, six in five RSS modules, 14 in 5 NOE modules and 14 in 6 NOS modules. The differential expression analysis can capture only differentially expressed genes (DE genes) between inter-group comparison, for instance, two parental strains of BXD RI strains (i.e., C57BL/6J and DBA/2J) in the Baker’s study. In contrast, network-based co-expression analysis is able to detect the population-level differentially expressed genes, even for minor changes.

One example of connecting genotype to phenotype is the differential regulation of histone genes relative to our experimental conditions. In this study, we identified eight histone genes, including *Hist1h2af*, *Hist1h2ag*, *Hist1h2ah*, *Hist1h2ai*, *Hist1h2ak*, *Hist1h2an*, *Hist1h2ao* and *Hist2h2ac*, that were dysregulated under either stress- or alcohol-induced conditions (RSS, NOE and RSE) compared to control (NOS). This observation is consistent with a recent study that linked histone modification to alcohol exposure in BXD strains (van der Vaart et al., 2017). Histone modification plays a fundamental role in epigenetic regulation, influencing gene expression (Esteller, 2007). Histone modification is subjected to many post-translational modifications including acetylation and methylation. The effects of alcohol metabolism on histone acetylation have been demonstrated in animal experiments (Albaugh et al., 2011).

Here, we have been able to identify genes that are affected under specific conditions (exposure to stress, alcohol, or their combination) as well as the preservation of modules across conditions (i.e., genes were generally dysregulated in one condition or another, but not multiple conditions). We have also identified genes that are not only co-expressed, but connected at the protein level. Finally, we were able to show that some modules and their associated expression patterns were correlated with the respective stress- and alcohol-related phenotypes. These findings are indicative of shared molecular function and provide insight into how these genes might affect phenotypes of interest. For example, subnetwork 1 included GABA receptor genes, GABA decarboxylase enzyme genes, and GABA synapse genes. This suggests more than the general involvement of GABAergic signaling, but rather that several components of GABAergic signaling are involved. More specifically, this finding suggests that at least six GABA receptor subtypes, two enzymes related to the synthesis of GABA, and several genes that are part of GABA synapse functioning are involved in alcohol-related behaviors including dependence, locomotion, preference, and withdrawal severity (Buck et al., 1997; Phillips et al., 1998; Demarest et al., 1999). Similarly, subnetwork 3 contains genes that are associated with glutamate signaling and includes both AMPA and NMDA receptor genes.

The integrative analysis we have used and demonstrated here, combined with other powerful and emerging resources will aid in future examinations and understanding of molecular networks affecting phenotypes. For example, the BXD RI panel represents a powerful genetic resource which, as shown here, has been important in studying stress, alcohol and its interaction. Importantly, the progenitors of this panel C57BL/6J (B6) and DBA/2J (D2) differ for both alcohol- and stress-related traits. The B6 strain exhibits a high level of alcohol consumption, whereas the D2 strain shows avoidance (De Waele et al., 1992). In contrast, the D2 strain is more vulnerable to stress than the B6 strain (Mozhui et al., 2010). Many other alcohol- and stress-related phenotypes are available for the BXD mice in the GeneNetwork database^4^. Perhaps more importantly, both parental strains have been sequenced, containing about 5 million sequence variants (Wang et al., 2016). Therefore, we should be able to identify sequence variants for any of alcohol- and stress-related traits acquired in the BXD mice. Emerging resources include “omics” data such as proteomics and metabolomics. High-throughput proteomics and metabolomics data can be readily generated by liquid chromatography coupled with tandem mass spectrometry (LC-MS/MS). By combining proteomics and metabolomics with the integrative approach we have used here, we could provide more complete information on molecular networks involved in alcohol and stress responses. Moreover, single-cell RNA sequencing and single cell-type proteomics are also emerging. With these technologies, we could potentially identify the major cell types involved in alcohol- and stress-responses.

The hippocampus is a key region that helps mediate response to stressors. A hallmark is feedback inhibition of the HPA axis. The hippocampus is also particularly vulnerable to the effects of alcohol. Smith et al. (2016) found that the hippocampus is the most affected brain region by chronic ethanol using co-expression analysis of time-course expression data in mice. Both human and rodent studies have shown that prolonged exposure to both stress and alcohol leads to hippocampal atrophy (Redila et al., 2006). The hippocampus is an especially suitable site to study the interaction of stressors and alcohol. In this study, we further dissected the hippocampus into nine specific cell types using single cell RNAseq data, and found that four cell types (i. e. endothelial, astrocyte, ependymal and microglia) show statistically enriched in different modules. The Astrocytes are the most enriched cell type in co-expressed modules. Astrocytes are star-shaped glial cells, playing an important role in the normal functioning of the central nerve system. Recently, several studies demonstrated that astrocyte structural plasticity (such as astrocyte protrusion length, branching and volume) is disrupted after long-term exposure to stress, which may be the underlying mechanism of stress-induced anxiety behaviors (Mayhew et al., 2015; Bender et al., 2016). Another possible explanation is the close interaction between astrocyte-derived peptides and receptors involved in the control of anxiety-related behaviors, such as benzodiazepine receptors, which are also localized on astrocytes (Rao et al., 2001).

The sample size is an important factor for constructing a robust network. A minimum sample size is 20, which is recommended by WGCNA program^5^. In our study, we use 41 strains (138 samples) for a combined data. Moreover, we used strain means (an average of female and male), which reduced the variance that could cause false correlations and reduce network robustness. A recent report published by Hoffman et al. (2018) suggested that increase in sample size would not yield a systematic difference in the number of co-expression modules as long as the number of strains exceeds the minimum number (i.e., 20). To evaluate whether our generated modules are robust, we performed module preservation analysis for female and male strains from NOE condition. The result showed that all 27 modules showed preserved with Z summary >2, and four modules exhibit Z summary <10. A simulation study (Langfelder et al., 2011) suggested that Z summary >10 indicates strong evidence that the module is preserved, and 2 < Z summary <10 indicates weak to moderate evidence of preservation. Thus, we believe that the strain number used in this study is sufficient to generate robust modules (Supplementary Figure S7).

While gene co-expression network approach has been successfully applied in a variety of studies related alcohol consumption (Vanderlinden et al., 2013; Saba et al., 2015; Hoffman et al., 2018), withdrawal (Iancu et al., 2013; Metten et al., 2014), and dependence (Ponomarev et al., 2012; Farris et al., 2015), there are computational and biological limitations in using co-expression for gene functional inference. First, it typically requires a large sample size (e.g., *n* ≥ 20) as discussed above. Second, co-expressed gene modules with similar expression patterns may not necessarily have related functions. Third, co-expressed gene modules sometimes rely on parameters used in WGCNA, such as minimum module size and the power β used as a soft threshold. Finally, expression patterns may not exhibit co-expression for some genes that do have related functions due to post-transcriptional regulation.

In summary, by integrating gene co-expression network, PPI network, and causality analysis, we provide evidence that alcohol and stress responses are regulated by functional modules. This study also underscores the importance of integrative analyses for identifying genes involved in addiction research. Our application of this integrative approach offers new insight into the molecular networks underlying essentially any phenotype or complex trait of interest.

## Author Contributions

XW contributed to the conception and design of the project. LL, MNC and BCJ contributed to the experiments. JL, XW, PX, PC, SX, GW, HW and XL analyzed and interpreted the data. XW, JL, LL, MNC and BCJ wrote the manuscript.

## Conflict of Interest Statement

The authors declare that the research was conducted in the absence of any commercial or financial relationships that could be construed as a potential conflict of interest.

## Footnotes

^1^http://www.illumina.com/

^2^www.genenetwork.org

^3^http://www.genenetwork.org/webqtl/main.py?FormID=annotation

^4^www.genenetwork.org

^5^https://labs.genetics.ucla.edu/horvath/CoexpressionNetwork/Rpackages/WGCNA/faq.html
