# Supplementary material for: Integrating Genetic and Gene Co-expression Analysis Identifies Gene Networks Involved in Alcohol and Stress Responses
Source: Front Mol Neurosci. 2018 Apr 5;11:102. doi: 10.3389/fnmol.2018.00102 (PMC5895640; doi:10.3389/fnmol.2018.00102)
Supplement: Supplementary file 1 [file Data_Sheet_1.doc]

**Supplementary Information**

**for**

Integrating Genetic and Co-expression Analysis Identifies Gene Networks Involved in Alcohol and Stress Response

*Jie Luo, Pei Xu, Peijian Cao, Hongjian Wan, Xiaonan Lv, Shengchun Xu, Gangjun Wang, Melloni N. Cook, Byron C. Jones, Lu Lu, Xusheng Wang*

Table of Contents

[Figure S1. Correlation of expression between replicates across 4 conditions. 2](#__RefHeading___Toc504859799)

[Figure S2. Differentially expressed genes detected from each condition 3](#__RefHeading___Toc504859800)

[Figure S3. Left side: The soft thresholding index R2 (y-axis) as a function of different powers β (x-axis). Right side The mean connectivity (y-axis) is a strictly decreasing function of the power β (x-axis). 4](#__RefHeading___Toc504859801)

[Figure S4. Co-expression analysis of four individual datasets. 5](#__RefHeading___Toc504859802)

[Figure S5. Heat maps of Pearson correlation and p value between modules and traits for 4 conditions. 6](#__RefHeading___Toc504859803)

[Figure S6. Module preservation and specificity between NOE and NOS, RSE and NOE, RSE and NOS, as well as RSS and NOS. 7](#__RefHeading___Toc504859804)

[Figure S7. Module preservation and specificity between male and female strains from NOE condition. 8](#__RefHeading___Toc504859805)


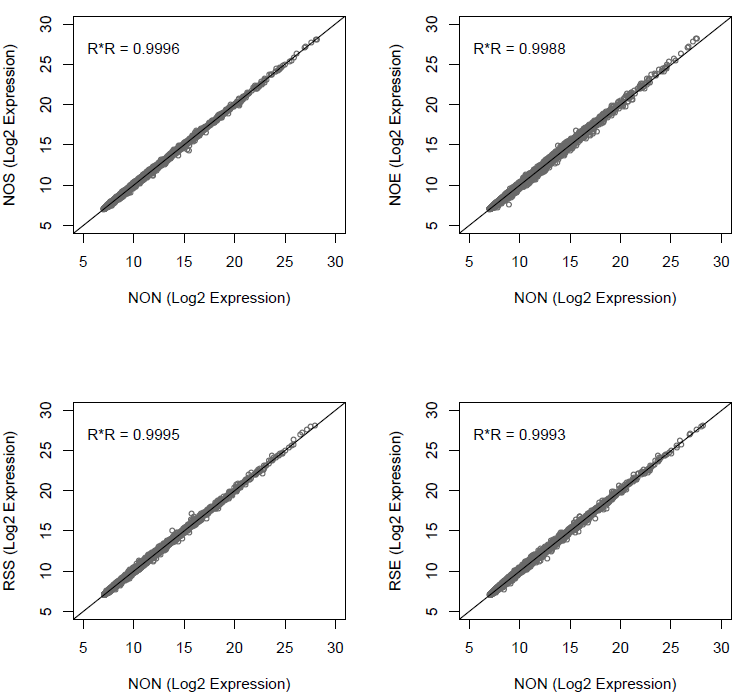


Figure S1. Correlation of expression between replicates across 4 conditions. The expression values are log2 transformed. The analysis shows a high correlation between each replicate.


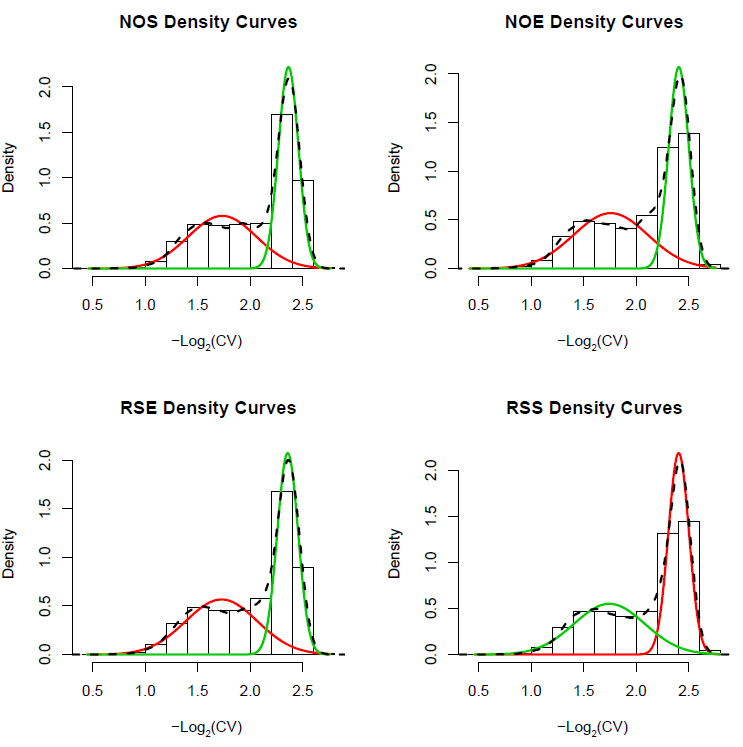


Figure S2. Differentially expressed genes detected from each condition. Variation in gene expression, measured by the coefficient of variation (CV), was calculated for each gene across BXD RI strains. The distributions of CV were fitted by two normal distributions using the EM algorithm.


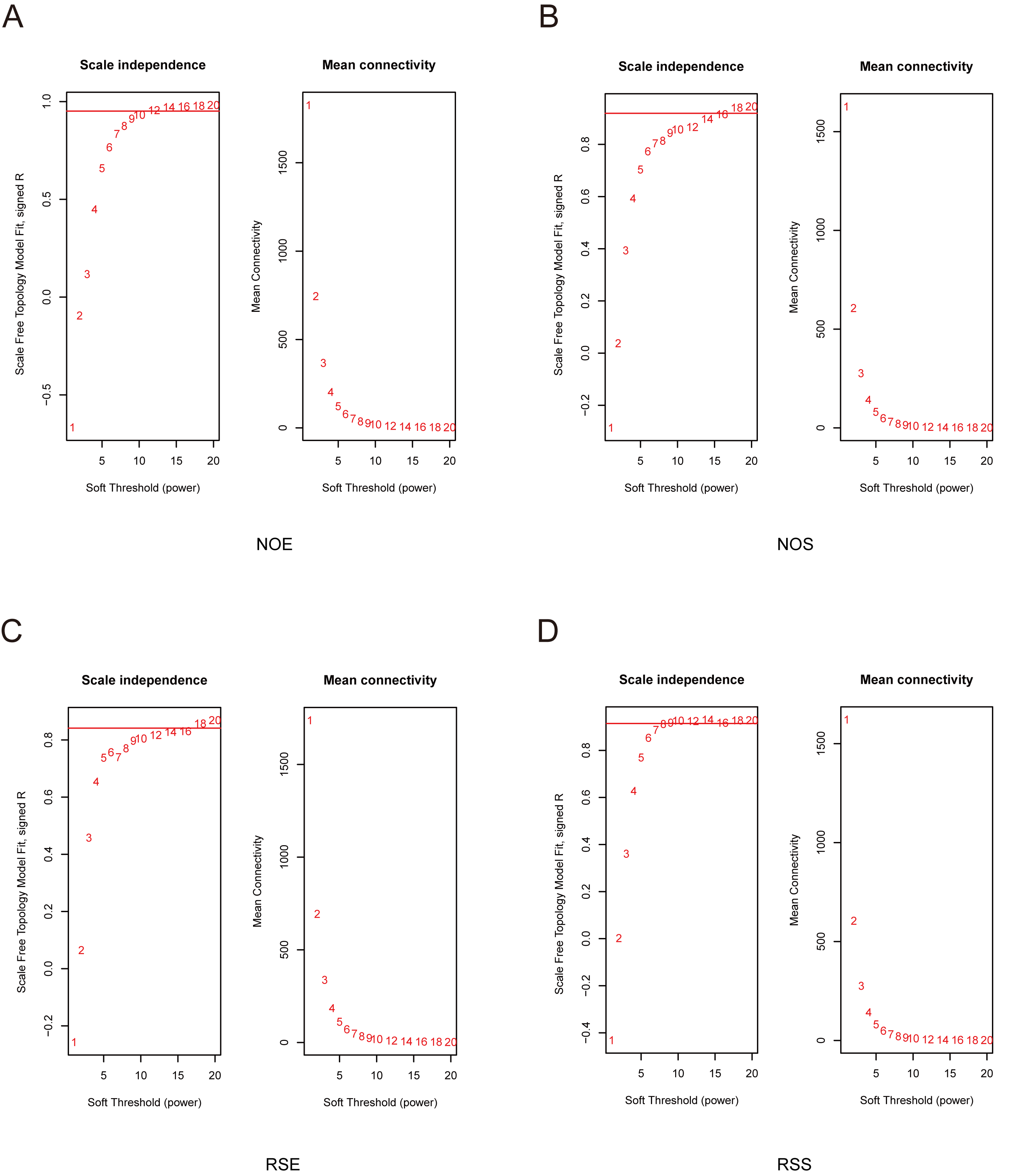


Figure S3. Determination of the soft thresholding power. Leftside: The soft thresholding index R^2^ (y-axis) as a function of different powers β (x-axis). Rightside: The mean connectivity (y-axis) is a strictly decreasing function of the power β (x-axis). The soft thresholding index and mean connectivity for (A) NOE; (B) NOS; (C) RSE; (D) RSS.


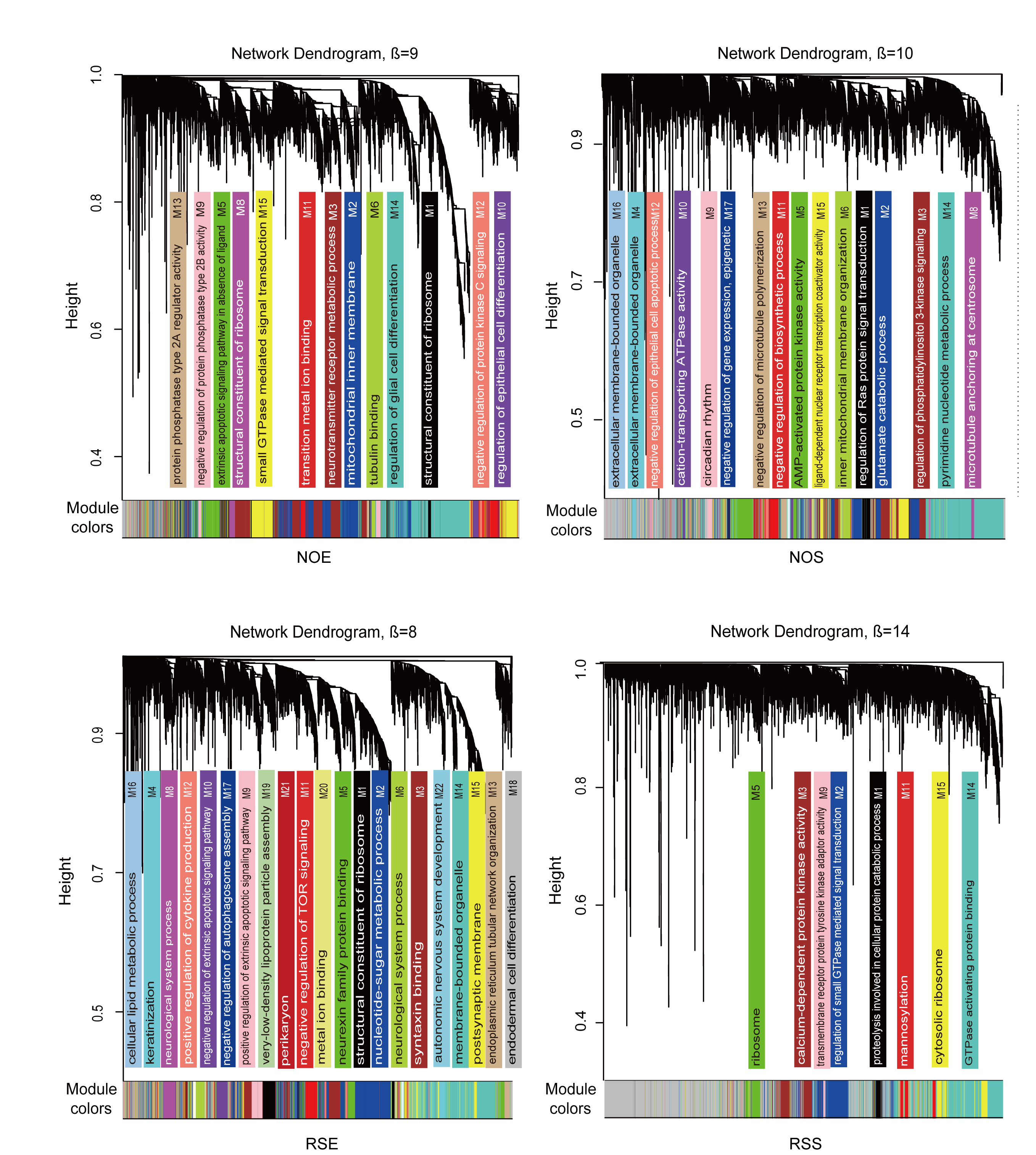


Figure S4. Co-expression analysis of four individual datasets. The analysis procedures are the same as Figure 2.


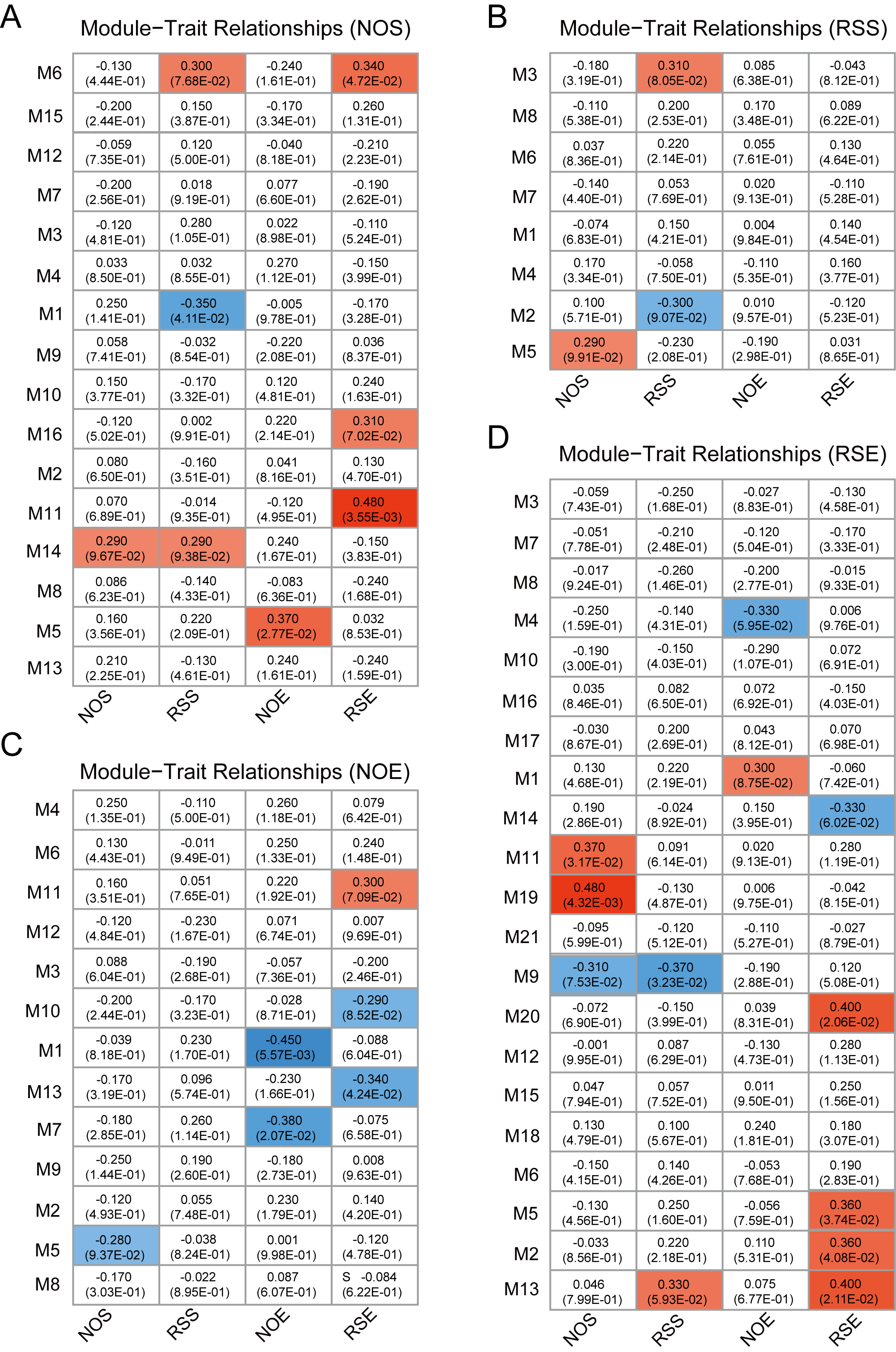


Figure S5. Heat maps of Pearson correlation and p value between modules and traits for 4 conditions. Each cell represents the correlation coefficient (and *p* value) computing from correlating module eigengenes (rows) to traits (columns). Only those correlations with |*p*| < 0.1 are shown. Correlation coefficients and *p* values for (A) NOS; (B) RSS; (C) NOE; (D) RSE.


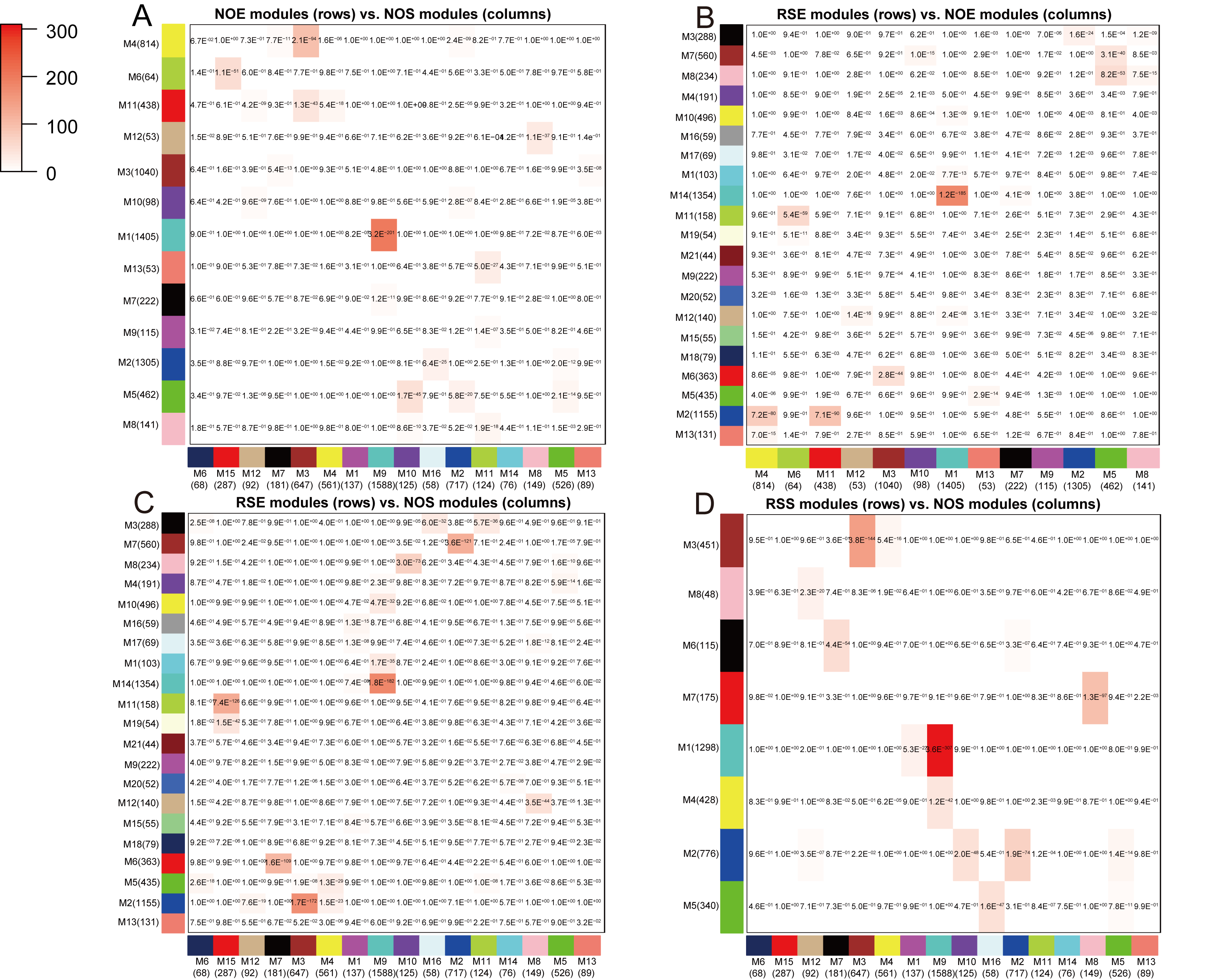


## Figure S6. Module preservation and specificity between (A) NOE and NOS, (B) RSE and NOE, (C) RSE and NOS, as well as (D) RSS and NOS.


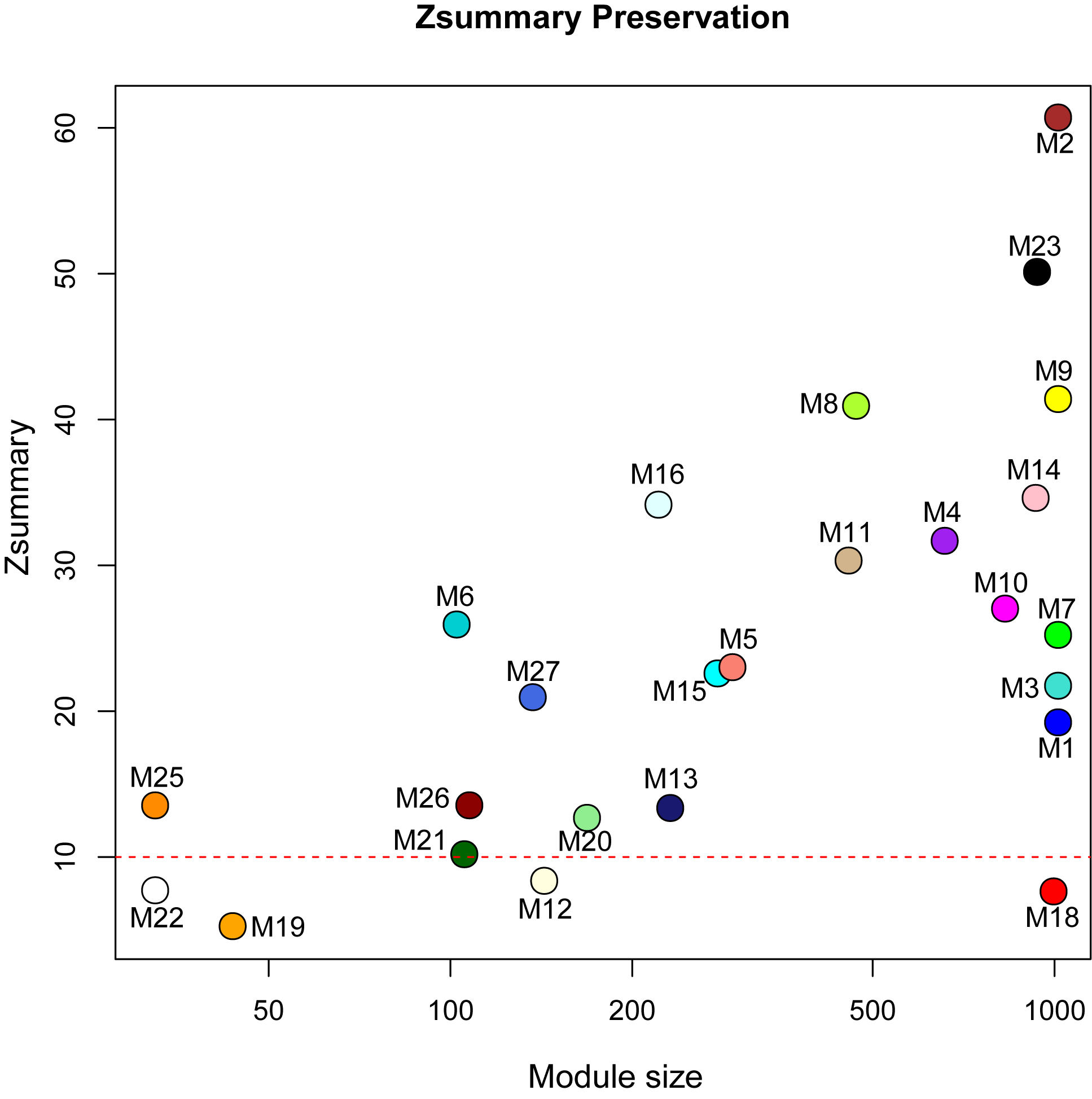


## Figure S7. Module preservation and specificity between male and female strains from NOE condition.
